# CBX4 Regulates Replicative Senescence of WI-38 Fibroblasts

**DOI:** 10.1155/2022/5503575

**Published:** 2022-02-23

**Authors:** Yu-Hsiu Chen, Xin Zhang, Kuei-Yueh Ko, Ming-Feng Hsueh, Virginia Byers Kraus

**Affiliations:** ^1^Duke Molecular Physiology Institute, Duke University, Durham, NC, USA; ^2^Division of Rheumatology/Immunology/Allergy, Department of Internal Medicine, Tri-Service General Hospital, National Defense Medical Center, Taiwan; ^3^Department of Pathology, Duke University Medical Center, Durham, NC, USA; ^4^Department of Orthopaedic Surgery, Duke University, Durham, NC, USA; ^5^Department of Biostatistics & Bioinformatics, Duke University, Durham, NC, USA; ^6^Department of Medicine, Duke University School of Medicine, Durham, NC, USA

## Abstract

Cellular senescence is characterized by cell cycle arrest and senescence-associated secretory phenotypes. Cellular senescence can be caused by various stress stimuli such as DNA damage, oxidative stress, and telomere attrition and is related to several chronic diseases, including atherosclerosis, Alzheimer's disease, and osteoarthritis. Chromobox homolog 4 (CBX4) has been shown to alleviate cellular senescence in human mesenchymal stem cells and is considered a possible target for senomorphic treatment. Here, we explored whether CBX4 expression is associated with replicative senescence in WI-38 fibroblasts, a classic human senescence model system. We also examined whether and how regulation of CBX4 modifies the senescence phenotype and functions as an antisenescence target in WI-38. During the serial culture of the WI-38 primary fibroblast cell line to a senescent state, we found increased expression of senescence markers, including senescence *β*-galactosidase (SA-*β*gal) activity, protein expression of p16, p21, and DPP4, and decreased proliferation marker EdU; moreover, CBX4 protein expression declined. With knockdown of CBX4, SA-*β*gal activity and p16 protein expression increased, and EdU decreased. With the activation of CBX4, SA-*β*gal activity, p16, and DPP4 protein decreased. In addition, CBX4 knockdown increased, while CBX4 activation decreased, gene expression of both CDKN2A (encoding the p16 protein) and DPP4. Genes related to DNA damage and cell cycle pathways were regulated by CBX4. These results demonstrate that CBX4 can regulate replicative senescence in a manner consistent with a senomorphic agent.

## 1. Introduction

Cellular senescence is a state of permanent cell cycle arrest related to telomere attrition, DNA damage, chronic inflammation, mitochondrial dysfunction, or other causes [1]. Cellular senescence has long been proposed as an anticancer mechanism since it can prevent the proliferation of cells with genomic instability [2]. It has also been linked to the pathogenesis of several chronic diseases, including atherosclerosis, Alzheimer's disease, and osteoarthritis (OA) [1, 3],. Cellular senescence was first described by Hayflick and Moorhead in 1961 as the phenomenon of cessation of cell division of human WI-38 fibroblasts after a maximum of 50 cell cycles [4]. The WI-38 primary cell line, originating from fetal lung tissue, has been widely used in vaccine development [5] and the study of senescence [6].

Senescent cells are characterized by decreasing proliferation and increasing cell granularity, cell size, lysosome content, and senescence-associated secretory phenotypes (SASPs). The accumulation of senescent cells and their secretion of SASPs are considered risk factors for age-related diseases. Several markers have been associated with cellular senescence, including increased senescence *β*-galactosidase (SA-*β*-gal) activity [6], and increased expression of p16 protein (gene CDKN2A), p21 protein (gene CDKN1A) [7], DPP4 protein (also known as CD26) [8], and decreased Lamin B1 protein (gene LMNB1) [9] and proliferation marker EdU [10]. These markers are generally used to identify cellular senescence and could be used to monitor the effects of senolytic agents to eliminate senescent cells and/or senomorphics to modify senescence phenotypes.

Targeted senomorphic strategies that preserve senescent cells but eliminate their detrimental effects might preserve tissue function and reserve better than senolytic strategies. For this reason, our goal was to investigate one possible agent, chromobox homolog 4 (CBX4), for senomorphic characteristics. CBX4 is a nuclear protein detected in all cells. CBX4 has been shown to alleviate cellular senescence in human mesenchymal stem cells (hMSCs) and to attenuate OA upon local overexpression in an experimental posttraumatic OA mouse model [11]. CBX4, a polycomb repressive complex (PRC1) associated protein and an E3 small ubiquitin-related modifier-protein(SUMO) ligase [12], has been discovered to regulate protein activity involved in DNA damage repair [13]. Higher expression of CBX4 has been implicated in the progression of hepatocellular cancer, breast cancer, and osteosarcoma [14], [15], [16] but has a protective effect in colon cancer [17]. CBX4 has also been shown to regulate cell proliferation, differentiation, and self-renewal in hematopoietic stem cells and epidermal stem cells [18, 19]. However, CBX4 expression and its role in cellular senescence in a terminally differentiated cell, such as WI-38 fibroblasts, have not been thoroughly investigated. To fill this knowledge gap, we investigated the role of CBX4 in replicative senescence in WI-38 human diploid lung fibroblasts. We hypothesized that CBX4 could regulate WI-38 replicative senescence, preserve tissue integrity by reducing inflammation and maintaining cell viability, and thereby function as a senomorphic agent. We confirmed the senomorphic capability of CBX4 through modification of WI-38 senescence phenotypes with gain and loss of CBX4 expression *in vitro*.

## 2. Materials and Methods

### 2.1. Cell Line Culture

The human diploid fibroblast WI-38 cell line (American Type Culture Collection, ATCC® CCL75™) was cultured in Minimum Essential Medium Eagle (Sigma-Aldrich, M4655) containing 10% FBS (Sigma-Aldrich, F2442), 1× Non-Essential Amino Acids Solution (Gibco, 11140050), 1 : 100 L-Glutamine–Penicillin–Streptomycin solution (Sigma-Aldrich, G6784), and maintained in a 37°C, 5% CO2 incubator. WI-38 fibroblasts were cultured from a cumulative population doubling (CPD) level of 37 representing a proliferative state based on Sidler et al. [20, 21], to a senescent state defined by a failure to double after one week (CPD 56-60) [6]. Serial culture of WI-38 was performed by seeding cells at a density of 7000 cells/cm^2^ and trypsinization with 0.25% (weight/volume) Trypsin-0.53 mM EDTA solution every 3-4 days until senescent status was reached. Cell numbers were recorded for estimated CPD and doubling time. Doubling time was calculated as follows: Doubling time = duration∗log_2_ (2)/log_2_ (Final Cell Concentration)–log_2_ (Initial Cell Concentration) [22].

### 2.2. Lentiviral Transduction of CBX4

Lentiviral transduction was done without adding polybrene, which could induce cellular senescence [23]. GFP expressing lentiviral particles (copGFP, Santa Cruz Bio, sc-108084) were used as a control to monitor and optimize transduction efficiency. The copGFP control lentiviral particles added at MOI 0.5-8 to WI-38 with puromycin selection for 4 days were able to achieve >90% stable GFP expression (Figure S1). As our outcomes of interest related to senescence can be induced by stress, our goal was to maximize transduction efficiency with a minimum of stress. For this reason, for these studies, we elected to avoid the use of polybrene that we observed caused some cytotoxicity in this model system. CBX4 knockdown (KD) experiments were performed with CBX4 shRNA and control shRNA lentiviral particles (Santa Cruz Bio, sc-38193-V, sc108080). CBX4 shRNA and control lentiviral particles were added to presenescent WI-38 (CPD47-50) at a multiplicity of infection (MOI) of 0.5, 1, and 2. Culture media were changed 24 hours after transduction. 3 days later, transduced cells were trypsinized and reseeded at a density of 7000/cm^2^ in 24-well plates. Puromycin 0.5 *μ*g/ml was added to the culture medium to select stably transduced cells. After 3-4 days of selection, CBX4 KD cells were collected for further assays. CBX4 activation (ACT) experiments were performed with a CBX4 CRISPR/Cas9 Synergistic Activation Mediator (SAM) system and control particles (Santa Cruz Bio.sc-403903-LAC, sc-437282). CBX4 activation and control lentiviral particles were added to presenescent WI-38 (CPD47-50) at a MOI 6. Culture media were changed 24 hours after transduction. 3 days later, transduced cells were trypsinized and reseeded at a density of 7000 cells/cm^2^ in 24-well plates. Antibiotics (puromycin 0.5 *μ*g/ml, blasticidin 1 *μ*g/ml, and hygromycin 50 *μ*g/ml) were added sequentially to select stably transduced cells. After blasticidin selection, cells were detached and reseeded at a density of 7000 cells/cm^2^ in 24-well plates and cultures continued with hygromycin selection. After 15-17 days of antibiotics selection, CBX4 ACT cells were collected for further assays.

### 2.3. Senescence *β*-Galactosidase (SA-*β*-gal)

CellEvent Senescence Green Flow Cytometry Assay Kit (Thermo Fisher, C10840) was used to quantify SA-*β*-gal [24] activity. Following cell collection, 1 − 2 × 10^4^ cells were fixed with 100 *μ*l 4% paraformaldehyde (PFA, Thermo Fisher 50980487) for 15 minutes, washed twice with phosphate-buffered saline (PBS) containing 1% bovine serum albumin (BSA, Sigma A3294), and incubated with senescence green working solution, 100 *μ*l at 37°C for 2 hours. Next, cells were washed twice with PBS containing 1% BSA, with detection using an Attune NxT flow cytometer (Thermo Fisher) or followed by permeabilization for costaining with EdU or p16. Unstained cells were used to determine the fluorescence background.

### 2.4. EdU Cell Proliferation Assays

EdU [10] (5-ethynyl-2′-deoxyuridine) (EMD Millipore, 1710525; Thermo Fisher, C10424) at a concentration of 10 *μ*M was added to the WI-38 cell culture media for 24 hours. Cells were harvested and counted; 1 − 2 × 10^4^ cells were fixed with 4% PFA and permeabilized with permeabilization buffer (Thermo Fisher, 00-8333-56), then incubated with Click working solution for 30 minutes. Finally, cells were washed twice with permeabilization buffer, followed by detection with an Attune NxT for flow cytometer. Unstained cells were used to determine the fluorescence background.

### 2.5. Flow Cytometry

The staining method for flow cytometry was done as previously described [25]. Following cell collection, 1 − 2 × 10^4^ cells were resuspended in 100 *μ*l PBS containing 1% BSA. PE-conjugated anti-DPP4 mAb (2 : 100, Thermo Fisher, 12-0269-42) was added for 30 mins at room temperature. After surface staining, the cells were washed, followed by flow cytometry acquisition or fixation and permeabilization for intracellular staining. For p21 staining, WI-38 cells were incubated with AF488-conjugated anti-p21 mAbs (1 : 100, CST, 5487). Unstained cells were used to determine the fluorescence background. For p16 staining, WI-38 cells were stained with unconjugated anti-p16 mAbs or a control antibody (20 : 100, both from Roche CINtec kit 9517) for 30 mins; then followed by AF647-conjugated anti-mouse IgG2a secondary antibody (1 : 1000, Jackson, 115607186). The stained cells were analyzed using an Attune NxT flow cytometer. Data were analyzed using FlowJo V10.8 software (BD Life Sciences) and were presented as gated percentage.

### 2.6. Western Blot

WI-38 cells were lysed using RIPA lysis buffer (Sigma-Aldrich, R0278) or a PARIS™ Kit (Thermo Fisher, AM1921) with a protease inhibitor cocktail (Sigma-Aldrich, P8340). PARIS™ Kit was used in lentiviral transduced samples to enable the use of cell lysates for both RNA and protein detection. A total of 5-10 *μ*g extracted protein, determined by the DC protein assay (Bio-Rad, 5000112), was mixed with 4× Laemmli sample buffer (Bio-Rad, 1610747), heated to 95°C for 5 minutes, and electrophoresed (100 V for 2 hours) on 10% Mini-PROTEAN® TGX Stain-Free™ Protein Gels (Bio-Rad, 4568033). Proteins were transferred onto polyvinylidene fluoride (PVDF) membranes using the Trans-blot turbo system (Bio-Rad, 1704274), using a standard SD protocol: up to 1.0 A; 25 V for 30 minutes followed by blocking with Tris-buffered saline Tween 20 buffer (TBST, Thermo Fisher, 28360) containing 5% fat-free milk (CST, 9999 s) for 1 hour at room temperature. Membranes were washed three times with TBST and then incubated with primary antibodies, anti-CBX4 mAb (1 : 1000, CST, 30559), and reference control anti-*β*-actin-HRP mAb (1 : 2000, Santa Cruz Bio, sc-47778 HRP) and made in TBST containing 5% BSA with incubation overnight at 4°C. Membranes were washed three times with TBST, incubated with anti-rabbit IgG-HRP (1 : 500, Thermo Fisher, 32460) at room temperature for 1 hour, and then washed three times using TBST. *β*-Actin protein bands were visualized using Clarity™ Western ECL Substrate (Bio-Rad, 1705060); CBX4 protein bands were visualized using SuperSignal™ West Pico PLUS Chemiluminescent Substrate (Thermo Fishers, 34579). Membrane images were acquired with the ChemiDoc XRS system (Bio-Rad). Grey band density values were analyzed and calculated using Image lab (version 6.0, Bio-Rad).

### 2.7. Quantitative Real-Time Polymerase Chain Reaction (qRT-PCR)

RNA was extracted using an Aurum™ Total RNA Mini Kit (Bio-Rad, 7326820) or PARIS™ Kit (Thermo Fisher, AM1921). cDNA was synthesized using the iScript™ cDNA Synthesis Kit (Bio-Rad, 1708891). Subsequently, qRT-PCR was performed using a SYBR green master mix (Applied Biosystems, 4309155) with a QuantStudio 6 Flex Real-Time PCR System (Applied Biosystems). Gene expression of *CDKN1A*, *CDKN2A*, *DPP4*, *LMNB1*, and *CBX4* was measured with *YWHAZ* as an internal reference control gene (see Table S1 for a list of primers).

### 2.8. qPCR Array

A custom RT2 Profiler PCR Array (Qiagen, 330171) was used to profile a total of 42 genes related to cellular senescence and/or CBX4 [1, 13, 26], along with 3 house-keeping genes, 1 genomic DNA control, 1 reverse transcription control, and 1 positive PCR control (Table S2). cDNA was synthesized using an RT^2^ First Strand Kit (Qiagen, 330404). Subsequently, qRT-PCR was performed using a RT^2^ SYBR Green ROX qPCR Mastermix (Qiagen, 330522) with the QuantStudio 6 Flex Real-Time PCR System (Applied Biosystems). The CT value of each gene was normalized with reference gene YWHAZ, ΔCT = CT (target gene) − CT (YWHAZ), and relative gene expression change in serial culture was calculated relative to the earliest passage (CPD earliest) from each serial culture experiment, ΔΔCT = ΔCT(a target sample) − ΔCT(CPD earliest); fold change (FC) = 2^−ΔΔCT^ was expressed using Log2 FC.

### 2.9. Statistical Analysis

Data are presented as mean ± standard error of the mean (SEM). Analyses were performed using GraphPad Prism 9 (GraphPad software) and R Statistical Software (manufacturer). To test the correlation coefficient between senescence markers and gene expression across the range of CPD from the serial culture and from the biological replicates, we used repeated measures correlation (Rmcorr) [27]; the repeated measures correlation coefficients (*r*_rm_) are presented with a 95% confidence interval (CI). In addition, paired *t*-tests were used to compare data from control vs. knockdown or activation of CBX4. A mixed-effects model was used to compare the knockdown effects of CBX4 shRNA with MOI 0.5, 1, and 2. *p* < 0.05 was considered statistically significant.

### 2.10. Protein-Protein Interaction Network

STRING (Search Tool for the Retrieval of Interacting Genes/Proteins) V11.5 [28] was used to evaluate the known and predicted biological relationships of the qPCR array genes and corresponding proteins interactions; interactions with high confidence (score > 0.7) are reported. Gene Ontology (GO) enrichment analysis was performed.

### 2.11. Ingenuity Pathway Analysis (QIAGEN IPA)

Gene expression of CBX4 ACT and CBX4 KD was normalized to the corresponding controls and analyzed by the QIAGEN IPA platform (QIAGEN Inc., https://digitalinsights.qiagen.com/IPA). IPA core analysis was used to assess canonical pathway enrichment for CBX4 ACT and KD separately. Bio-functional pathways regulated by CBX4 ACT and CBX4 KD were compared in a clustered hierarchical heat map.

## 3. Results

### 3.1. Senescence Markers Were Upregulated in the Senescent WI-38 Cells

**Figure 1:**
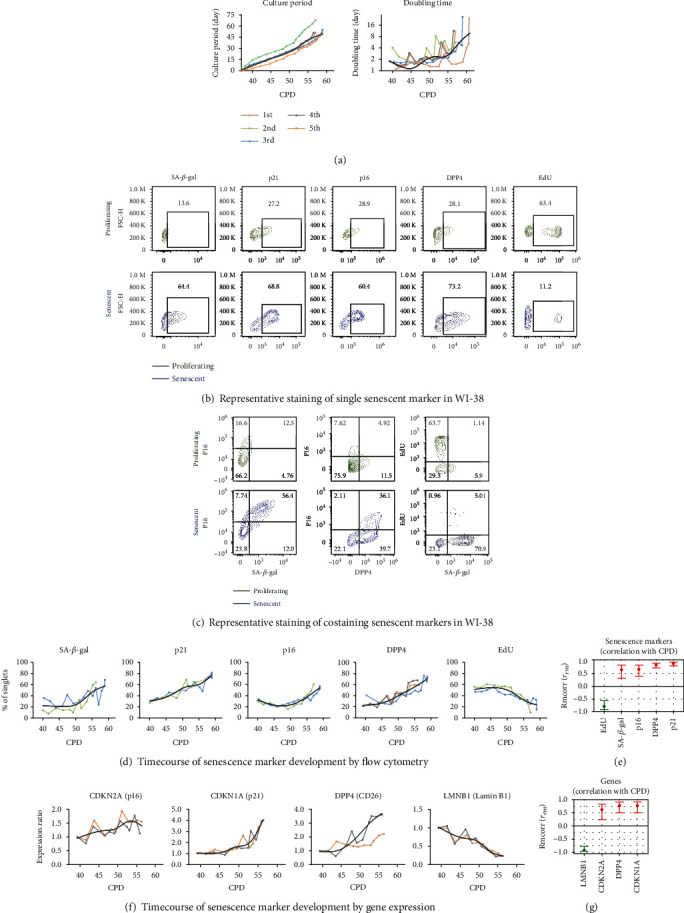
The timecourse of senescence marker expression with the serial culture of the WI-38 replicative senescence cell model system. Human diploid fibroblast WI-38 cells were profiled for senescence markers by flow cytometry and qPCR. Each serial culture is represented in a different color; the trend-line for expression of each marker was created by a smoothing spline function with 5 knots. (a) Serial culture of WI-38 was performed from CPD 37 to senescent status (CPD 56–60) for a total of 5 times. Doubling time increased during the serial culture. (b) and (c) SA-*β*-gal activity, protein expression of p21, p16, DPP4, and EdU proliferation markers detected by flow cytometry in proliferating (CPD 40-42, dark green) and senescent (CPD 56-60, blue) WI-38, both individually as single markers (b) or combined (c). Compared to proliferating WI-38, senescent WI-38 expressed higher SA-*β*-gal activity, p21, p16, DPP4 protein, and lower EdU (b). In the senescent WI-38, p16 protein was positively co-expressed with SA-*β*-gal activity and DPP4; SA-*β*-gal activity was negatively correlated with the proliferation marker EdU (c). (d) and (f) Serial culture of WI-38 from proliferating to senescent status was done repeatedly to characterize the timecourse of senescence marker development by flow cytometry and qPCR. There are no qPCR equivalents for SA-*β*-gal activity or EdU; however, Lamin B1 (LMNB1) was quantified by qPCR to provide a gene expression representation of cell proliferation and senescence. (e) and (g) The associations of senescence markers and genes with CPD were evaluated by repeated measures correlation (Rmcorr). The Rmcorr correlation efficient (*r*_rm_) of CPD with senescence markers (% of total cells) and genes (expression ratio) is depicted for analyses adjusted for repeated measurements. Green: negative correlation. Red: positive correlation.

Five independent serial cultures of WI-38 fibroblasts were performed from CPD 37 through CPD 60 when senescent status was attained (Figure 1(a)). The culture period from seeding (CPD 37) to senescent status (a failure to double after one week) was 54.80 ± 3.44 days; the average CPD corresponding to WI-38 senescence was 58.04 ± 0.78 CPD. We observed increased doubling time during the serial culture (Figure 1(a)). Flow cytometry was used to quantify SA-*β*-gal activity, protein expression of p21, p16, and DPP4 (CD26), and the proliferation marker, EdU. Compared to proliferating WI-38 (CPD 40-42), senescent WI-38 (CPD 55-60) expressed higher SA-*β*-gal activity and protein expression of p21, p16, DPP4, and lower proliferation marker EdU (Figure 1(b)). The p16 protein was coexpressed with SA-*β*-gal activity and DPP4 in senescent WI-38 cells (Figure 1(c)). As expected, EdU reflecting cell proliferation was greater in proliferating WI-38 than in senescent WI-38 and negatively correlated with SA-*β*-gal activity. During serial culture, SA-*β*-gal activity, and protein expression of p21, p16 and DPP4 increased, while EdU proliferation decreased (Figure 1(d)). CPD was positively correlated with SA-*β*-gal activity, protein expression of p21, p16, DPP4, and negatively correlated with EdU (Figure 1(e)). Interestingly, some markers changed relatively early in the course of the serial culture, such as p21 protein that increased from around CPD 40 and DPP4 protein that increased from CPD 45. Other markers changed later, such as p16 protein and SA-*β*-gal activity (all increased), and EdU proliferation (decreased) at around CPD 50 (Figures 1(a) and 1(d)). Gene expression with serial culture was consistent with protein level changes with an increase in senescence markers CDKN2A (p16), CDKN1A (p21), DPP4 (CD26), and a decrease in LMNB1 (Lamin B1), a gene expression measure of cell proliferation known to be decreased with senescence development [29] (Figure 1(f)). Similar to protein level changes with serial culture, CPD was positively correlated with gene expression of CDKN2A, CDKN1A, and DPP4 and negatively correlated with LMNB1 (Figure 1(g)).

### 3.2. CBX4 Decreased during WI-38 Serial Culture and Knockdown of CBX4 Increased Senescence

**Figure 2:**
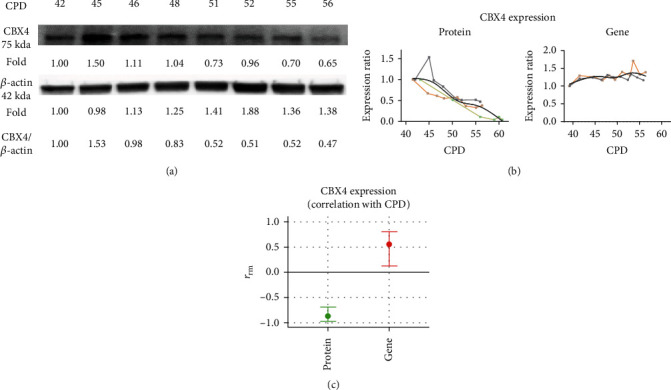
CBX4 decreased with WI-38 serial culture. CBX4 protein and gene expression were investigated in each generation of the WI-38 serial culture. Protein expression was examined by Western blot and normalized with *β*-actin. Gene expression was analyzed by qPCR and normalized with YWHAZ. Decreased CBX4 protein expression with serial culture is shown in a representative Western blot (a). CBX4 protein expression (*n* = 3) and gene expression (*n* = 2) in serial culture were normalized to controls and depicted as an expression ratio relative to CPD 41 ((b) left, protein) and CPD 39 ((b) right, gene). The Rmcorr correlation coefficient (rrm) and confidence interval of CPD with CBX4 protein and gene expression are depicted (c) for repeated measurements. Green: negative correlation. Red: positive correlation.

Given the reported role of CBX4 in regulating cellular senescence [11, 18], we next evaluated CBX4 protein expression and gene expression from proliferating through senescence status in the WI-38 cells. As shown in Figures 2(a)–2(c), CBX4 protein expression decreased significantly with serial passage (*r*_rm_ = 0.877, *p* < 0.001); in contrast, CBX4 gene expression increased with serial passage (*r*_rm_ = 0.555, *p* < 0.001). Moreover, compared to proliferating WI-38 (CPD 41), CBX4 protein decreased around 50% at CPD 50 (Figure 2(b)), when p16 and SA-*β*-gal activity started to increase and EdU proliferation decreased (Figure 1(d)). These results suggest that change in CBX4 protein may be involved in regulating replicative senescence.

**Figure 3:**
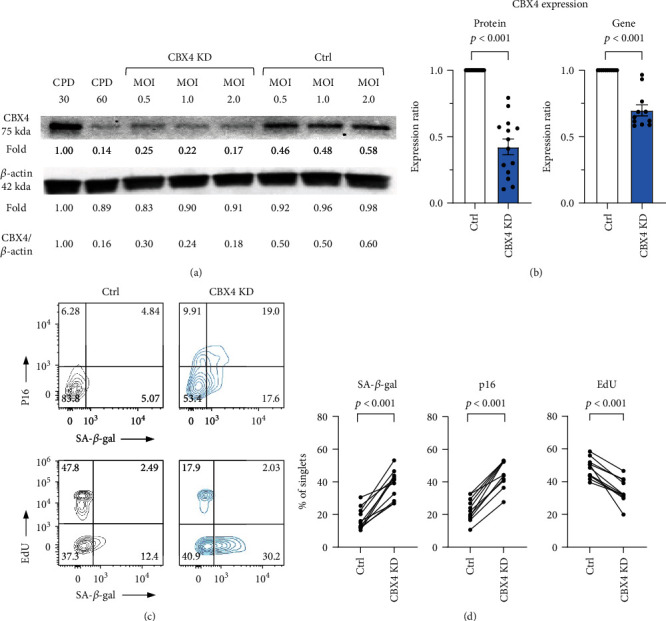
Knockdown of CBX4 increased senescence markers in WI-38 cells. Knockdown (KD) of CBX4 was performed with CBX4 shRNA carried by lentiviral particles with different multiplicity of infection (MOI) 0.5, 1, and 2. (a) Representative Western blot of CBX4 protein expression in proliferating (positive control CPD30) and senescent (negative control CPD60) WI-38, in CBX4 KD and lentiviral control (Ctrl) at three MOI. (b) Overall significant reduction of CBX4 protein (*n* = 14) and gene expression (*n* = 11) due to lentiviral KD (control grey, CBX4 KD light blue). (c) and (d) SA-*β*-gal activity, p16 and EdU proliferation expression in control and CBX4 KD in representative flow cytometry (C), and overall demonstrating increased protein expression of senescence markers SA-*β*-gal activity and p16, and decreased proliferation marker EdU after knockdown of CBX4 ((d), *n* = 11).

We next evaluated the effect of CBX4 knockdown in presenescent WI-38 passages (CPD 47-50). Compared to the control group, cell numbers were significantly lower in the CBX4 knockdown group (Figures S2A and B). Overall CBX4 protein and gene expression were significantly decreased in the CBX4 knockdown group: mean difference of protein expression ratio −0.577 ± 0.059; and mean difference of gene expression ratio −0.302 ± 0.041 (Figures 3(a) and 3(b), all *p* < 0.001). Similar levels of knockdown in CBX4 protein and gene expression were achieved with MOI 0.5, 1, and 2 (Figure S3A). After CBX4 knockdown, SA-*β*-gal activity increased 21.26% ± 2.60%, p16 protein increased 22.89% ± 1.81%, and EdU proliferation decreased 13.76% ± 1.88%, all significantly (Figures 2(c) and 2(d), all *p* < 0.001). Similar degrees of change in senescence markers were achieved with MOI 0.5, 1, and 2 (Figure S3B). Our findings suggest that a mean 57% knockdown of CBX4 protein was sufficient to cause a cellular senescent phenotype in WI-38. This result is consistent with our observation in serial cultures that most senescence markers developed at 50% reduction of CBX4 protein.

### 3.3. CBX4 Activation Decreased Senescence in WI-38

**Figure 4:**
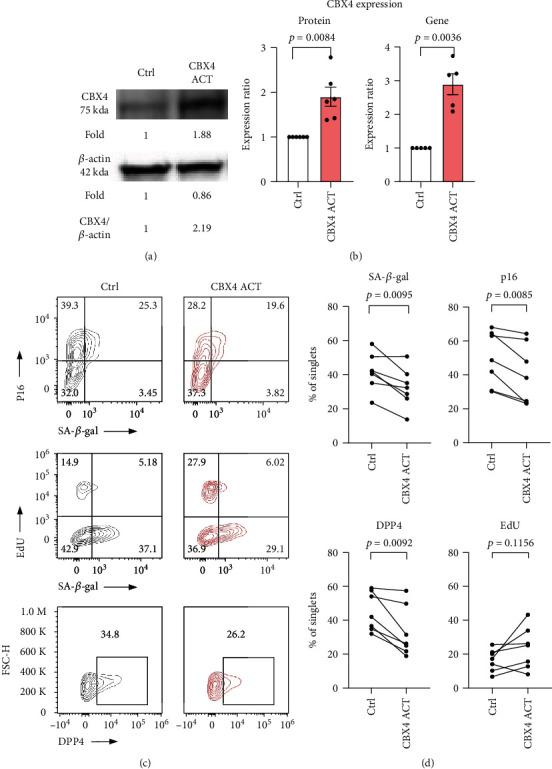
Activation of CBX4 reduced cellular senescence markers in WI-38. CBX4 was activated using the CRISPR/Cas9 Synergistic Activation Mediator (SAM) lentiviral particle system in presenescent WI-38 (CPD47-50). (a) Representative Western blot of CBX4 protein expression in CBX4 activation (ACT) and lentiviral control (Ctrl). (c) CBX4 protein (*n* = 6) and gene expression (*n* = 5) both increased in response to CBX4 ACT compared with Ctrl (Ctrl: white, CBX4 ACT: light red). (c) SA-*β*gal, p16, and EdU proliferation expression in Ctrl and CBX4 ACT in representative flow cytometry. (d) By flow cytometry, CBX4 activation resulted in decreased protein expression of senescence markers SA-*β*gal, p16, and DPP4 and nonsignificantly increased EdU (*n* = 7).

Based on these results, we hypothesized that activation of CBX4 would be senomorphic and decrease senescence in the WI-38 cells. We activated endogenous CBX4 expression using the CRISPR/Cas9 Synergistic Activation Mediator (SAM) system. Compared to control, cell numbers were not significantly different in the CBX4 activation group (Figures S2C and D). With this activation system, both increased CBX4 protein and gene expression were achieved: protein mean difference ratio + 0.901 ± 0.214, *p* = 0.0084; and gene mean difference ratio + 1.894 ± 0.310, *p* = 0.0036 (Figures 4(a) and 4(b)). In association with CBX4 activation, p16 and DPP4 protein expression and SA-*β*-gal activity were decreased as profiled by flow cytometry: p16 − 9.25% ± 2.41%, *p* = 0.0085; DPP4 − 12.24% ± 3.24%, *p* = 0.0092; and SA − *β* − gal activity − 9.27% ± 2.47%, *p* = 0.0095 (Figures 4(c) and 4(d)). However, the mean percentage of EdU positive cells was not significantly increased: +7.11% ± 3.87%, *p* = 0.116. Our results are consistent with a senomorphic effect of CBX4 in WI-38 cells.

### 3.4. Senescence-Related Genes and Pathways Regulated by CBX4

**Figure 5:**
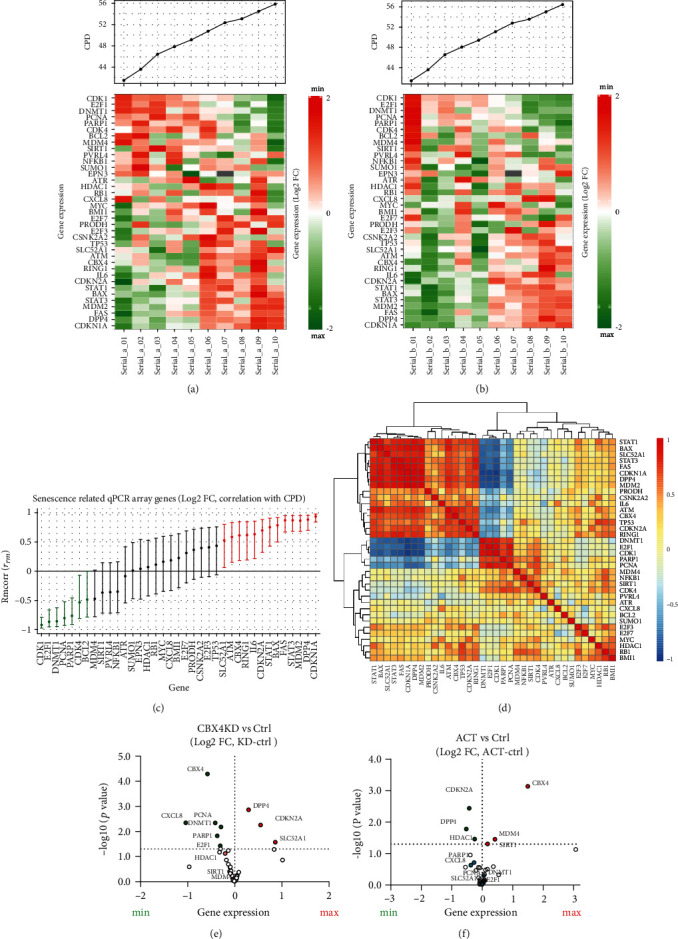
Regulation of senescence-related genes by CBX4. A selected panel of senescence-related genes was analyzed by qPCR microarray. Two serial culture experiments were performed, and the resulting Log2FC values were computed (normalized with YWHAZ and calculated relative to the earliest CPD of serial culture). (a) and (b) Heatmaps of standardized gene expression at 10 time points of WI-38 serial culture performed in two biological replicates. (c) the Rmcorr correlation (and 95% confidence intervals) of CPD with expression (Log2 FC) of 37 senescence-related genes from serial culture. Green: positive correlation of gene expression with CPD. Red: negative correlation gene expression with CPD. Black: insignificant correlation of gene expression with CPD. (d) Heatmap of pairwise Spearman correlations of genes measured in the serial culture experiment. Based on expression pattern and consistent with (a) and (b), two groups of genes were highly clustered. (e) Volcano plot of gene expression changes in response to CBX4 KD vs. Ctrl showing increased expression of DPP4, CDKN2A, and SLC52A1 and decreased expression of CXCL8, PCNA, DNMT1, E2F1, and PARP1. (f) Volcano plot of gene expression changes in response to CBX4 ACT vs. Ctrl showing decreased expression of DPP4, CDKN2A, and HDAC1 and increased expression of CBX4, SIRT1, and MDM4. Red: increased expression, green: decreased expression.

We examined the gene changes during WI-38 cell serial culture from proliferating to senescent cell state (11 time points) by qPCR array. A total of 37 out of 42 selected genes were detected; DAO, TP63, TNF, LY6D, and SOX2 were unable to be detected. Relative gene expression results were ordered in a heat map based on correlation with CPD values (Figures 5(a) and 5(b)). With serial culture, expression of several genes (such as CDK1, E2F1, DNMT1, PCNA, and BCL2) decreased, while other genes (such as CDKN1A, DPP4, FAS, and MDM2) increased. A total of 20 of the 37 detected genes were significantly correlated with CPD (Figure 5(c)). Pairwise correlations among the genes revealed that two groups of genes strongly clustered together (Figure 5(d)). The first group consisted of STAT1, BAX, SLC52A1, STAT3, FAS, CDKN1A, DPP4, and MDM2 whose expression increased with increasing CPD of serial culture. In contrast, the second group consisted of DNMT1, E2F1, CDK1, PARP1, and PCNA whose expression decreased during serial culture. Subsequently, we examined changes in gene expression in response to knockdown and activation of CBX4 compared to their paired control. Knockdown of CBX4 decreased expression of CXCL8, PCNA, DNMT1, E2F1, and PARP1 and increased expression of DPP4, SLC52A1, and CDKN2A (Figure 5(e)). Activation of CBX4 in presenescent cells increased expression of SIRT1 and MDM4 and decreased expression of DPP4, HDAC1, and CDKN2A (Figure 5(f)). Interestingly, CBX4 knockdown increased, while CBX4 activation decreased, gene expression of both CDKN2A and DPP4, suggesting that these genes may be direct targets and mediators of CBX4 regulation of senescence.

**Figure 6:**
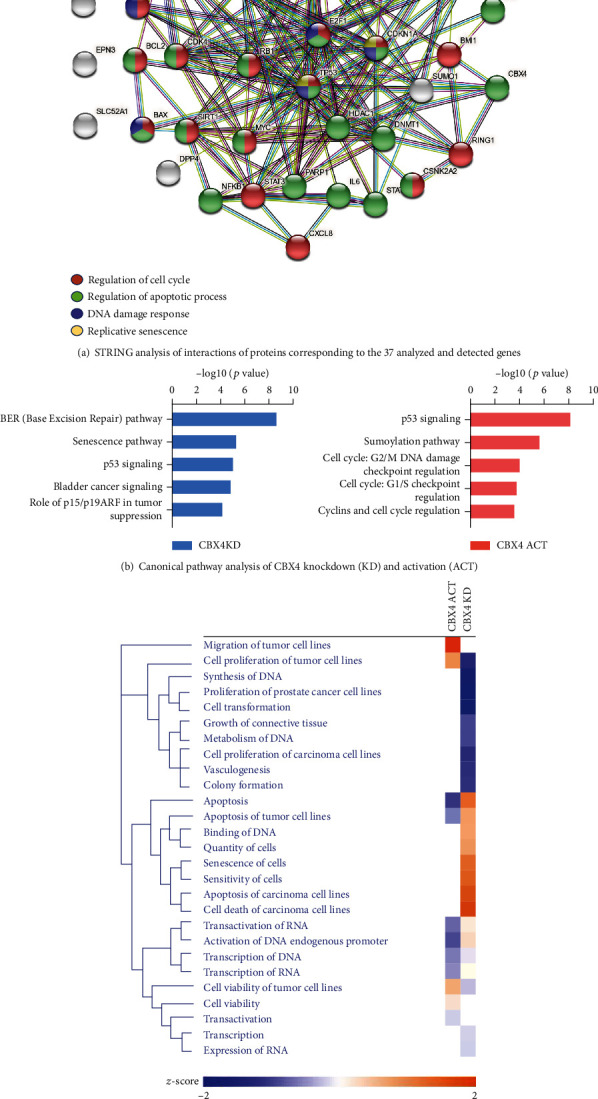
String network and IPA pathway analysis of CBX4 activation and knockdown in senescence. (a) STRING analysis of interactions of proteins corresponding to the 37 analyzed and detected genes. Red: genes related to regulation of cell cycle (GO: 0051726, strength: 1.0, FDR: 1.57e-15); green: genes related to regulation of apoptotic process (GO: 0042981, strength: 0.91, FDR: 8.16e-15); purple: genes related to DNA damage response, signal transduction by p53 class mediator resulting in cell cycle arrest (GO: 0006977, strength: 1.95, FDR: 4.18e-14); yellow: proteins related to replicative senescence (GO: 0090399, strength: 2.31, FDR: 2.15e-08). (b) and (c) CBX4 knockdown (KD, light blue) and activation (ACT, light red) were normalized with the control for the gene expression and analyzed for canonical pathways (b) and compared using biofunction pathway analysis and presented in the heat map ((c), orange: increase; blue: decrease).

The protein-protein interactions (PPI) of the 37 proteins corresponding to the 37 detected genes were analyzed with STRING. STRING identified 37 nodes, 160 edges, 8.65 average node degrees, an average local clustering coefficient of 0.604, and PPI enrichment *p* value < 1.0e-16; taken together, this indicates our selected panel represented a highly interactive network. The Gene Ontology (GO) showed our detected panel associated with several senescence-related pathways such as DNA damage repair, cell cycle, apoptosis, and replicative senescence (Figure 6(a)). IPA core analysis identified several canonical pathways related to CBX4 regulation including DNA damage repair, cell cycle regulation, and p53 signaling pathway; each of these senescence-related pathways was involved in CBX4 knockdown and activation (Figure 6(b)). Hierarchical cluster heatmaps based on biofunctional pathways demonstrated that cellular proliferation and cell viability were higher, and apoptosis, transcription of RNA and DNA were lower with CBX4 ACT compared with CBX4 KD (Figure 6(c)).

## 4. Discussion

In this study, we investigated the role of CBX4 in replicative senescence using the human primary diploid fibroblast WI-38 model system. We characterized the senescence phenotype using multiple senescence markers, including population doubling time, p21, p16, SA-*β*-gal activity, DPP4, and proliferation marker EdU in serial culture. CBX4 protein expression decreased significantly during the serial culture of WI-38. By knockdown, we achieved a 57% reduction of CBX4 expression in presenescent WI-38, analogous to increasing the senescence phenotype ~5 CPD (estimation based on SA-*β*-gal activity and p16 protein expression); by activation, we achieved a 90% elevation of CBX4 expression analogous to reducing senescence ~2 CPD. Specifically, at the molecular level, knockdown of CBX4 increased gene expression of CDKN2A and DPP4 and decreased CXCL8, PCNA, DNMT1, E2F1, and PARP1; activation of CBX4 increased gene expression of SIRT1 and MDM4 and decreased DPP4, HDAC1, and CDKN2A. Taken together, our results demonstrate that CBX4 regulates replicative senescence in WI-38 cells.

We showed that DPP4 is highly correlated with WI-38 replicative senescence and could be useful as a senescence treatment target and as a senescence biomarker. The protein and gene expressions of DPP4 were both significantly positively correlated with CPD. Also, DPP4 expression was regulated up and down by CBX4 knockdown and activation, respectively. DPP4 is a transmembrane glycoprotein that can also circulate in a soluble form in plasma. DPP4 has been known to regulate glucose metabolism by inactivation of GIP; both GLP-1 and DPP4 inhibitors have been used for type 2 diabetes mellitus (DM) treatment [8]. DPP4 was recently identified as a surface marker on senescent human WI-38 primary fibroblasts and found to be more highly expressed on the surface human peripheral blood mononuclear cells isolated from individuals aged 78 to 88 yrs old compared to individuals aged 27 to 36 yrs old [8]. The cell surface expression of DPP4 makes it a promising candidate for targeted treatment of senescent cells through antibody-dependent cell-mediated cytotoxicity [8]. Treatment of senescence-related chronic disease, as shown by recent studies with DPP4 inhibitors that ameliorated atherosclerosis in type 2 DM patients [30, 31], prevented vascular aging [32] and protected chondrocytes from TNF-*α*-induced senescence [33]. We noticed increased SIRT1 in association with DPP4 reduction upon CBX4 activation of WI-38 cells. These results are consistent with a recent study showing that DPP4 inhibition reduced endothelial senescence by activating the AMPK/SIRT1/Nrf2 pathway [34]. Nevertheless, a complete understanding of the interrelationship of CBX4 and DPP4 remains to be elucidated.

We observed increased SA-*β*-gal activity, protein expression of p16 and p21, and decreased EdU proliferation with WI-38 serial culture. Although these results are consistent with previous studies in the WI-38 senescent model system [6, 8, 35], there was an interesting discordance in the temporal patterns of expression of the various senescence markers. For instance, we observed that decreased CBX4 protein expression in WI-38 serial cultures preceded the appearance of many senescence markers; thus, CBX4 could be a factor regulating WI-38 replicative senescence. This hypothesis is supported by the ability of CBX4 knockdown and activation to regulate the expression of many senescence-related genes, including HDAC1 shown to mediate the transition to a senescent phenotype [36]. This hypothesis is also supported by recent data from hMSCs showing that CBX4 deficiency leads to characteristics associated with premature cellular senescence, while CBX4 overexpression reduced these senescent markers including SA-*β*-gal activity, p21, and p16 [11]. In addition, we observed that serial cell passage of WI-38 led to increased expression of p21 earlier than p16. This observation is consistent with a prior study showing early regulation of p21 in a senescent fibroblast model [37]. It has therefore been suggested that p16, whose expression increases later than p21, may be critical in maintaining senescence status [37]. In contrast, p21 was recently shown to be related to immunosurveillance of senescent cells, mediated by p21 binding to pRB leading to cell cycle arrest and CXCL14 expression; clearance of the stressed cells by immune cells ensued if the p21 levels did not recuperate [38]. The fact that CBX4 knockdown and activation were not mirror-images of each other is also likely a result of a different CPD starting point for each of these manipulations since, as noted here, development of senescence in the WI-38 was not a linear process but rather a staged process with different markers having different inflection points in the process. Taken together, these data suggest that different senescence markers may predominate at different biological ages and that optimal monitoring of antisenescence treatment effects requires the use of markers appropriate to the stage of senescence being treated.

CBX4 is a member of the polycomb chromobox (CBX) family. CBX proteins, including CBX1/2/3/4/5/6/7/8, are major regulators of histone methylation, which can function as epigenetic modulators, maintaining heterochromatin organization and reducing related gene expression [39]. CBX proteins are known to be essential for cell proliferation, maintenance of adult stem cell populations, and regulation of stem cell self-renewal [39, 40]. Loss of CBX2 causes senescence-associated chromosomal rearrangements in mouse embryonic fibroblasts [41]. CBX7 regulates replicative senescence [42] and maintains pluripotency in embryonic stem cells and hematopoietic stem cells [19, 43]. CBX2/4/6/7/8 is involved in PRC-mediated inhibition of p16 expression [44]. Based on our data, a variety of mechanisms mediated by CBX4 may be involved in its senomorphic activities. For instance, the function of CBX4 SUMO E3 ligase activity has been shown to be associated with DNA damage repair mediated by CBX4 SUMOylation of BMI1 that stabilizes BMI1 on the DNA damage site and thereby facilitates DNA damage repair [13]. Also, CBX4 is a PRC1-associated protein; PRC1 has been shown to regulate cell cycle and gene transcription by chromatin modification [45, 46]. PRC1 was shown to bind the *p16* promoter and repress p16 expression in young cells [45]. This function is consistent with the increased p16 expression we observed upon CBX4 knockdown. Moreover, Ren et al. showed that CBX4 alleviates senescence in hMSCs, in part at least, by maintaining nucleolar homeostasis through repression of rRNA transcription. In our IPA analysis, we also found CBX4 activation decreased transcription of RNA pathways compared to CBX4 knockdown. Therefore, transcriptional repression by CBX4 may also contribute to the senomorphic effect.

Interestingly, activation of CBX4 led to a decrease in senescence markers and an increase in *SIRT1*, an important antagonist of the oxidative stress pathway; it surprisingly did not lead to a significant increase in cell proliferation. This may be due to the fact that CBX4 activation also leads to a decrease in HDAC1. HDAC1 inhibition was previously shown to increase p21 and decrease proliferation of WI-38 [47]. So, effects on HDAC1 may be counteracting possible oncogenic, pro-proliferative responses of WI-38 to CBX4. This interpretation is consistent with data showing that CBX4 interacted with HDAC1 to repress the tumor suppressor KLF6 in clear cell renal carcinoma while knockdown of HDAC1 restored KLF6 function [48]. These results may explain why activation of CBX4 did not significantly increase WI-38 proliferation in our study. Not surprisingly, one of the potential concerns related to CBX4 treatment is its oncogenic properties in hepatic cancer and breast cancer [14, 15]. However, activation of CBX4 in the terminal differentiated presenescent WI-38 fibroblast did not increase the proliferation of the cells, which may make CBX4 a good senomorphic target for aging, particularly if administered judiciously and with appropriate monitoring.

Although CBX4 gene and downstream protein expression were readily modulated at a transcriptional level in our knockdown and activation experiments with shRNA and the dCas9 system, respectively, we were surprised to observe a discordance between CBX4 gene (increase 1.5 times) and protein (significantly decreased) expression in the serial culture system. This might be explained by translational level regulatory mechanisms. Like many other transcriptional regulatory factors, CBX4 can be modulated by posttranslational modifications including conjugation to ubiquitin and ubiquitin-like proteins such as SUMO, that target CBX4 for degradation through the ubiquitin proteosome system [49] and by phosphorylation, methylation, and demethylation [50]. CBX4 itself is a SUMO E3 ligase so it is both sumoylated and sumoylates other proteins. In addition, SALL1 has been noted to enhance the stability of CBX4 protein by modulating its ubiquitination thereby avoiding its degradation via the proteasome [49]. Future analysis of SALL1 in the WI-38 model system might inform an understanding of the discordance in gene and protein expression of CBX4 with serial culture. Nevertheless, the dramatic decline in CBX4 protein and associated changes in WI-38 senescence cell phenotype with serial culture are fully consistent with results obtained with lentiviral modulation (repression and activation) of CBX4.

There were several limitations of this study. We were limited to evaluating WI-38 from CPD40 due to the lack of availability of very early passage numbers (<CPD30) from the ATCC. At the start of the serial cultures, there appeared to be a slight increase then decrease of senescence markers in cells immediately after thaw and culture; we attribute these perturbations to stress then recovery responses. We limited our study to replicative senescence, so results may not be applicable to other causes of senescence. We identified only 20 out of 42 genes correlated with CPD in the serial culture of WI-38. Additional genes associated with CPD might have been identified with a greater number of independent serial culture samples than the two we evaluated (corresponding to 22 total samples). In addition, Rmcorr analysis captures the linear relationship of gene expression and CPD; therefore, nonlinear dynamics of gene expression might not have been detected with this method. A strength of our study was the flow cytometry-based measurement that profiled senescence makers at the single cell level and discerned the associations of different senescence markers by their coexpression patterns. Another strength of this study was the use of the classic WI-38 model system, the first in which senescence was described [4], to explore the role of CBX4 in replicative senescence.

In summary, CBX4 protein expression decreased with serial culture of WI-38 cells. Knockdown of CBX4 increased cellular senescence, whereas activation of CBX4 decreased senescence. Notably, CBX4 activation was senomorphic; it was able to achieve a reduction in the senescence phenotype without cell killing or a marked increase in cell proliferation that might increase the risk of cancer. Based on the change in patterns of gene expression with CBX4 modulation, the mechanisms by which CBX4 may regulate senescence in WI-38 appear to be mostly related to the DNA damage repair pathway and PRC1-related cell cycle and transcriptional regulation. Taken together, our results demonstrate that CBX4 regulates replicative senescence in WI-38 cells therefore functions as a senomorphic and potential antisenescence target.

## Data Availability

All data generated or analyzed during this study are included in the published article.

## Supplementary Materials

Supplementary Materials All supplementary material is provided in the supplementary file. Table S1: primers used for RT-PCR detection of senescence target genes. Table S2: qPCR custom microarray panel (Qiagen, 330171). Figure S1: transduction efficiency of copGFP control lentiviral particles with different MOI and with/without adding polybrene. Figure S2: number of WI-38 cells in response to CBX4 knockdown (KD) or activation (ACT) compared to control (Ctrl). Figure S3: effect of CBX4 knockdown on senescence outcomes in WI-38 cells.
