# Supplementary material for: CBX4 Regulates Replicative Senescence of WI-38 Fibroblasts
Source: Oxid Med Cell Longev. 2022 Feb 23;2022:5503575. doi: 10.1155/2022/5503575 (PMC8890863; doi:10.1155/2022/5503575)
Supplement: Supplementary Materials — All supplementary material is provided in the supplementary file. Table S1: primers used for RT-PCR detection of senescence target genes. Table S2: qPCR custom microarray panel (Qiagen, 330171). Figure S1: transduction efficiency of copGFP control lentiviral particles with different MOI and with/without adding polybrene. Figure S2: number of WI-38 cells in response to CBX4 knockdown (KD) or activation (ACT) compared to control (Ctrl). Figure S3: effect of CBX4 knockdown on senescence outcomes in WI-38 cells. [file 5503575.f1.pdf]

Supplementary materials

CBX4 Regulates Replicative Senescence of WI-38 Fibroblasts

Yu-Hsiu Chen[1,2,6], Xin Zhang[1,3], Kuei-Yueh Ko[4], Ming-Feng Hsueh[1,3], Virginia Byers Kraus[1,3,5,6]

[1] Duke Molecular Physiology Institute, Duke University, Durham, NC, USA

[2] Division of Rheumatology/Immunology/Allergy, Department of Internal Medicine Tri-Service General Hospital, National Defense Medical Center

[3] Department of Orthopaedic Surgery, Duke University, Durham, NC, USA

[4] Department of Biostatistics & Bioinformatics, Duke University, Durham, NC, USA

[5] Department of Medicine, Duke University School of Medicine, Durham, NC, USA

[6] Department of Pathology, Duke University Medical Center, Durham, NC, USA

Corresponding Author: Virginia Byers Kraus

Address: 300 N Duke St, Durham, NC 27701

Email: vbk@duke.edu

Table S1. Primers used for RT-PCR detection of senescence target genes.

	F	R
CBX4	Qiagen, 330001 (PPH19160A)	
CDKN2A	Qiagen, 330001 (PPH00207C)	
YWHAZ	CTGAGGTTGCAGCTGGTGATGACA	AGCAGGCTTTCTCAGGGGAGTTCA
LMNB1	GAAAAAGACAACTCTCGTCGCA	GTAAGCACTGATTTCCATGTCCA
DPP4	AAAGGCACCTGGGAAGTCATCG	CAGCTCACAACTGAGGCATGTC
CDKN2A	GGACAGCAGAGGAAGACCAT	GGCGTTTGGAGTGGTAGAAA

Table S2. qPCR custom microarray panel (Qiagen, 330171)

**Gene Symbol**	**Assay Catalog #**	**Gene Symbol**	**Assay Catalog #**	**Gene Symbol**	**Assay Catalog #**
GDC	PPH65835A	PPC	PPX63339A	RTC	PPX63340A
B2M	PPH01094E	YWHAZ	PPH01017A	18SrRNA	PPH05666E
PVRL4	PPH09678B	PRODH	PPH00877A	LY6D	PPH19736C
DAO	PPH11264A	EPN3	PPH13321A	SLC52A1	PPH11141A
BAX	PPH00078B	BCL2	PPH00079B	MDM4	PPH00875E
MDM2	PPH00193E	FAS	PPH00141B	TP53	PPH00213F
TP63	PPH01032F	CDK1	PPH00116C	CDK4	PPH00118F
CDKN1A	PPH00211E	CDKN2A	PPH00207C	ATM	PPH00325C
STAT1	PPH00811C	STAT3	PPH00708F	NFKB1	PPH00204F
TNF	PPH00341F	IL6	PPH00560C	CXCL8	PPH00568A
HDAC1	PPH01735F	RB1	PPH00228F	E2F3	PPH00917F
E2F1	PPH00136G	E2F7	PPH19766A	SUMO1	PPH00973F
CSNK2A2	PPH02197F	DNMT1	PPH01055F	SOX2	PPH02471A
PARP1	PPH00686B	MYC	PPH00100B	RING1	PPH14334B
BMI1	PPH57778A	CBX4	PPH19160A	DPP4	PPH00035B
SIRT1	PPH02188A	PCNA	PPH00216B	ATR	PPH01318B

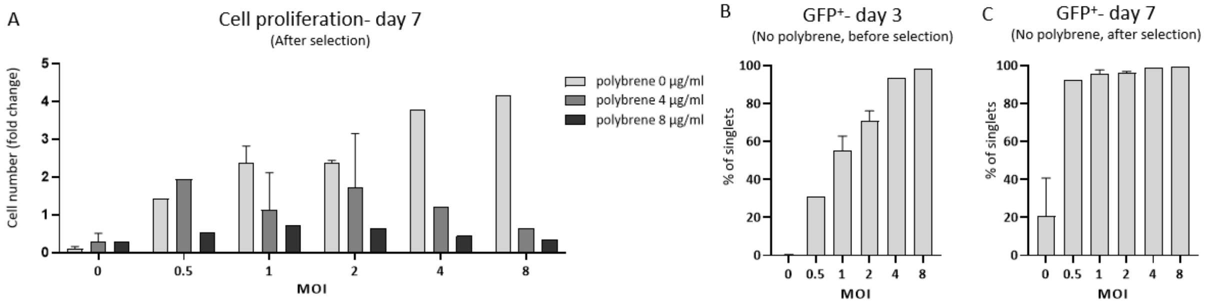

Figure S1.Transduction efficiency of copGFP control lentiviral particles with different MOI and with/without adding polybrene. A) Cell proliferation of WI-38 cells in response to the copGFP control lentiviral particles with and without adding polybrene. Cells numbers were measured by flow-cytometry; fold changes, computed as a ratio of day 7 harvested cell numbers to the day 3 seeded cell numbers, showed a cytotoxic effect of using polybrene comparing results for 0, 4 and 8 µg/ml polybrene. B) Without added polybrene, the percentage of GFP expressing cells determined by flow-cytometry three days after transduction at different multiplicity of infection (MOI) ranging from 0-8. C) Without added polybrene, the percentage of GFP expressing cells (determined by flow-cytometry) 7 days after transduction with puromycin 0.5 µg/ml selection for 4 days.

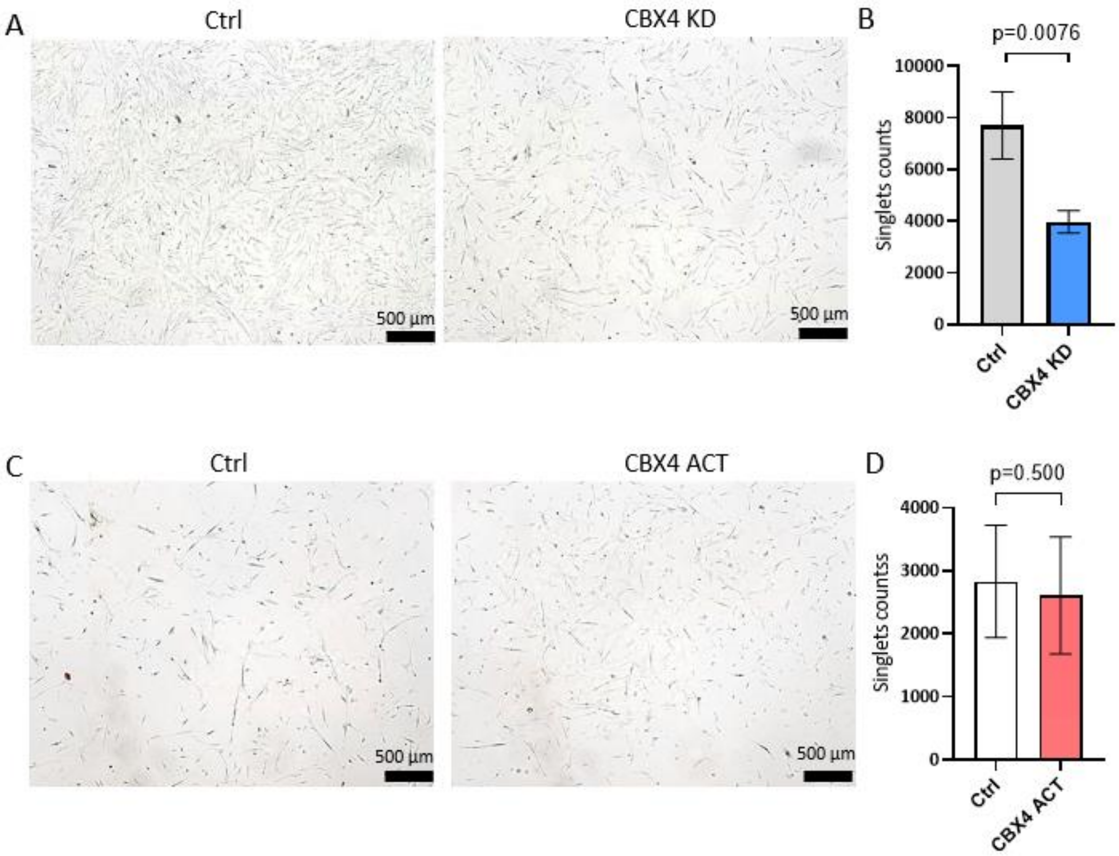

Figure S2. Number of WI-38 cells in response to CBX4 knockdown (KD) or activation (ACT) compared to control (Ctrl). A) Representative microscopic image of Ctrl and CBX4 KD in pre-senescent WI-38. B) Cell numbers were measured by flow-cytometric acquisition, gating singlets from 1 well/24 well plate (n=11, ctrl grey, CBX4 KD light blue). C) Representative microscopic image of Ctrl and CBX4 ACT in pre-senescent WI-38. D) Cell numbers were measured by flow-cytometric acquisition gating singlets from 1 well/24 well plate (n=7, ctrl white, CBX4 ACT light red).

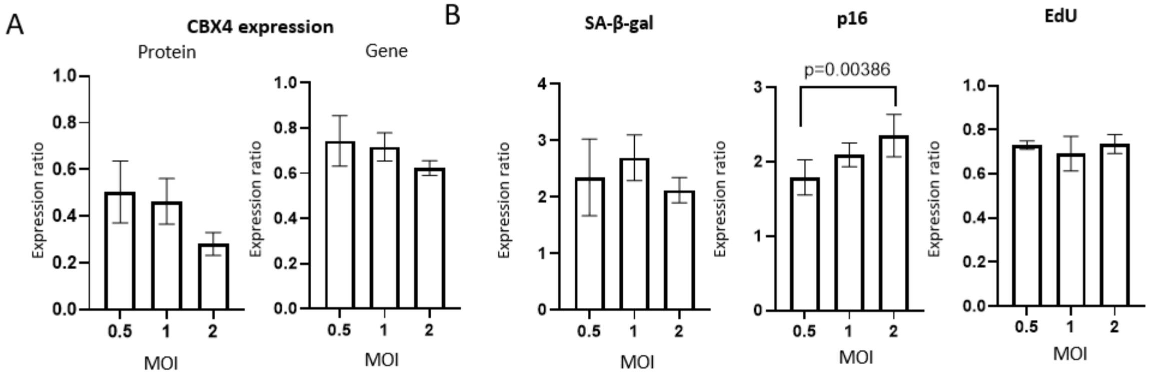

Figure S3. Effect of CBX4 knockdown on senescence outcomes in WI-38 cells. A) CBX4 protein and gene expression knockdown at different MOI by shRNA lentiviral particles at MOI 0.5, 1, and 2; protein n=4, 6 and 4 replicates, respectively (p=0.137 for difference by MOI) and gene n=3, 5 and 3 replicates, respectively (p=0.113 for replicates). B) Senescence markers were compared between MOI 0.5, 1 and 2 (n=3, 5 and 3, respectively): SA-βgal activity (p=0.550), and EdU (p=0.9746) did not show a significant difference by MOI dosage; p16 protein expression was higher at MOI 2 compared to MOI 0.5 (p=0.0386). A mixed-effects model was used to compare the effects of CBX4 knockdown.
